# Healthcare system responses to intimate partner violence in low and middle-income countries: evidence is growing and the challenges become clearer

**DOI:** 10.1186/s12916-017-0886-5

**Published:** 2017-07-12

**Authors:** Angela Taft, Manuela Colombini

**Affiliations:** 1 Judith Lumley Centre, La Trobe University, Bundoora, Melbourne, VIC Australia; 2 Department of Global Health and Development, London School of Hygiene and Tropical Medicine, 15-17 Tavistock Place, London, UK

**Keywords:** Intimate partner violence, Low and middle-income countries, Randomised controlled trials, Healthcare system, Health providers

## Abstract

The damage to health caused by intimate partner violence demands effective responses from healthcare providers and healthcare systems worldwide. To date, most evidence for the few existing, effective interventions in use comes from high-income countries. Gupta et al. provide rare evidence of a nurse-delivered intimate partner violence screening, supportive care and referral intervention from a large-scale randomised trial in Mexican public health clinics. No difference was found in the primary outcome of reduction in intimate partner violence. There were significant short-term benefits in safety planning and mental health (secondary outcomes) for women in the intervention arm, but these were not sustained.

This important study highlights the challenges of primary outcome choices in such studies, and further challenges for the sustainability of healthcare systems and healthcare provider interventions. These challenges include the role of theory for sustainability and the risk that baseline measures of intimate partner violence can wash out intervention effects. We emphasise the importance of studying the processes of adaptation, integration and coordination in the context of the wider healthcare system.

Please see related article: https://bmcmedicine.biomedcentral.com/articles/10.1186/s12916-017-0880-y

## Background

Violence against women, especially intimate partner violence (IPV), is a global pandemic. Evidence for the range of severe health impacts has galvanised national and international governments to include health system reform in the suite of policy responses to this form of violence [1]. However, to date, most of the rigorous studies underpinning our understanding of what works, or not, in low and middle-income (LMI) healthcare settings, have originated in high-income countries [2–4]. This is problematic, as the heaviest health burden is in LMI countries, where the capacity -whether human, financial or logistical - is very different [3, 5, 6].

## New evidence

A new study published in *BMC Medicine* [7] clearly identified the gap in evidence and the barriers that exist to conducting this form of pragmatic trial in LMI healthcare settings. It makes a significant contribution by illustrating the importance of and the challenges in generating rigorous evidence.

This cluster-randomised controlled trial of 42 large public health clinics in Mexico City [6] trained current nurses in both arms to integrate IPV screening into a general women’s health assessment. Nurses in the intervention arm were given further training to offer a more comprehensive response to screened women identified as experiencing IPV, including empathetic care, safety planning, harm reduction counselling and supportive referrals [6]. To those women identified as experiencing IPV after screening, nurses in the comparison arm offered a card to refer them to community-based support services.

The primary outcome of the study was to reduce IPV in the past year. The investigators included safety behaviours, challenging reproductive coercion, mental wellbeing and use of community resources as secondary outcomes. The study measured these pre-specified outcomes at three points, from baseline up to 15 months [6]. While there were some intermediate improvements in mental health and safety planning behaviours, women in both arms of the study reported reductions in IPV over time. The lack of a primary treatment effect at any time offers important lessons for overcoming challenges in IPV intervention studies in healthcare settings.

## Challenges of IPV study primary outcome choices

The study by Gupta et al. [8] raises a continuing question in pragmatic IPV trials about the choice and level of ambition of a primary outcome. This important study recognised the limitations of screening and identification strategies to increase referrals, consistent with current high quality evidence [5, 9]. The authors’ hypothesis was that if nurses screened, identified and counselled women, and provided safety plans and referrals to community resources, then their partner’s violence might decrease. However, this is likely to be out of the control of both the women and certainly nurses. Even direct advocacy services report modest reductions in violence by men against their partners [3]. The consistency with which screening trials increase identification but not referral suggests that healthcare providers need additional and repeat training, and resources to help manage those women who are unready or unwilling to be referred [5].

Studies of women’s behaviours over the many stages of their transition to safety [10] suggest that women’s ability to make changes depends on their self-efficacy, readiness to take action and desire to leave the relationship. Healthcare providers therefore need the skills and knowledge to identify a woman’s level of readiness to act, and to be taught a range of strategies if she is unready or unwilling to accept a referral. For many women this process may take well over 12 months, and it may take several years after receiving advocacy, depending on the level of abuse [3]. Currently we have insufficient evidence of effective healthcare provider training or specific strategies to help women experiencing IPV at each of the stages of change. The only exception to this result has been the IRIS study conducted in England by Feder et al. [10], in which advocates had a formal role in training and follow-up. Even so, compared with control patient groups, the absolute numbers of referred women in the intervention were smaller than the numbers of affected women who would have been in the overall patient population.

## Further methodological challenges

There is also an issue of bedevilling in IPV trials. Methodological considerations have encouraged investigators to measure the baseline level of violence in both arms of a study. Ethical guidelines have required them to offer modest support to identified women in the comparison arm. There is growing evidence that simply asking women about IPV can result in a Hawthorne effect; raising the level of consciousness of some women from pre-contemplation to contemplation, or even a higher level of action [11]. The investigators recognised that women identified as experiencing IPV in the comparison arm might have acted on the resources offered and sought help, thus attenuating the effect of the intervention. The need to investigate such impacts adds to the growing arguments for process evaluations to illuminate the aspects of a healthcare intervention that have the greatest effects on the outcome [4, 12].

Advancing this complex field of healthcare interventions also argues for longer-term follow-up of both women and providers to see whether there is sustained provider behaviour change and whether the ‘seed’ planted in the woman’s mind at the time of intervention might have germinated after 24 or 36 months (if not immediately). Gupta et al. recommend such work, which must also explore factors beyond the individual provider and the woman, and investigate the organisational and systems changes that may have been important at different levels of the healthcare system to support a wider and more integrated systems-wide response to IPV.

Interventions for IPV in healthcare settings are complex and require a sound theoretical basis to explain their hypotheses or logic models and to promote their implementation and sustainability. Healthcare providers work in complex systems and hierarchies, and specific adjustments may be required within the organisation and management structure to integrate IPV interventions and sustain changes in work practices [13, 14]. Coordinating and integrating strategies between and among all levels of a healthcare system are crucial to effective responses in health system interventions in LMI countries, irrespective of their entry point in primary or tertiary care [15]. Leaders, managers and advocates for improved responses to IPV are also important for enabling change [12]. Intervention development in LMI countries must also account for the presence or absence of ongoing structural support services outside the facility, including community-based resources, non-governmental organisations, legal frameworks and well-trained police. All of these are vital to offer long-term support and to understand the potential to maximise healthcare system and providers’ confidence and ability to support women [16].

## Conclusions

It is critical to understand the broader systems context in which healthcare interventions operate in LMI countries (which is relevant also for adaptation and scaling up), as the focus of previous studies has primarily been on the effectiveness of the intervention rather than the processes of adaptation, coordination and integration. We strongly recommend long-term intervention trials that follow women beyond the healthcare setting, to understand their coping mechanisms and use or non-use of the formal and informal support services available in their communities. As we have done in healthcare interventions in high income countries, we also recommend theory-driven investigation, including a strong focus on the sustainability of healthcare system responses and healthcare provider interventions in LMI countries [17, 18]. Comprehensive understanding of flexible, low-cost, high impact interventions in LMI countries is critical to ensure that the healthcare system is effective in improving the safety and lives of women and children in families exposed to IPV, and preventing their deaths.

## Abbreviations

IPV: Intimate partner violence
LMI: Low and middle-income
